# Dual benefits of *Bacillus velezensis* LJ-19: contact-dependent biocontrol of *Fusarium* wilt and growth promotion in cucumber

**DOI:** 10.3389/fpls.2025.1711383

**Published:** 2025-12-11

**Authors:** Songwei Li, Jingjing Li, Minghui Yuan, Jingxia Ren, Yang Jiao, Linjie Zhao, Yeyun Wang, Zihan Yan, JinYu Li, Jianfeng Du, Hongliang Wang, Chenyu Yang

**Affiliations:** School of Plant Protection and Environment (School of Bee Science), Henan Institute of Science and Technology, Xinxiang, Henan, China

**Keywords:** *Bacillus velezensis* LJ-19, *Fusarium* wilt, biocontrol, growth promotion, contact-dependent inhibition, induced systemic resistance

## Abstract

Cucumber is an important economic crop widely cultivated globally. *Fusarium* wilt, caused by *Fusarium oxysporum* f. sp. *cucumerinum*, seriously affects its yield and quality and is difficult to control. Here, we isolated a novel *Bacillus velezensisstrain* (LJ-19) from cucumber rhizosphere. Through assays for antibacterial activity, enzymatic activity, detection of disease resistance genes, and plant growth-promoting activity, this strain exhibited pronounced antagonistic activity against *Fusarium oxysporum* f. sp. *cucumerinum* and possessed plant growth-promoting traits. Notably, the inhibition of Fusarium oxysporum f. sp. cucumerinum mycelial growth and spore germination by LJ-19 was primarily contact-dependent, rather than mediated by diffusible antibiotics. Meanwhile, LJ-19 enhanced the activities of key defense enzymes—superoxide dismutase (SOD), peroxidase (POD), phenylalanine ammonia lyase (PAL), and polyphenol oxidase (PPO), thereby contributing to plant protection, and the transcript levels of defense-related genes, including Nonexpressor of pathogenesis-related genes 1 (NPR1), *Pathogenesis*-related gene 3 (PR3), *Lipoxygenase* 1 (LOX1), Constitutive triple response 1 (CTR1), and *Phenylalanine* ammonia-lyase 1 (PAL1), were up-regulated. Pot experiments demonstrated that LJ-19 treatment significant increased the stem thickness, fresh weight, leaf area, and overall biomass in cucumber. LJ-19 was also been confirmed could thrive in nitrogen-free environment and solubilize inorganic phosphorus, produce indole-3-acetic acid (IAA) and siderophores. Collectively, these findings demonstrate that *Bacillus velezensis* LJ-19 suppresses cucumber *Fusarium* wilt via direct antagonism and induction of systemic resistance. They also provide insights into the molecular mechanisms by which LJ-19 controls *Fusarium* Wilt and promotes cucumber growth, highlighting its potential as an effective biocontrol agent for sustainable cucumber.

## 1 Introduction

*Fusarium oxysporum* f. sp. *cucumerinum* is a soil-borne pathogen that causes cucumber wilt, posing a severe threat to cucumber production and leading to yield losses (Ali et al., 2022; Xu et al., 2023). In recent years, cucumber wilt has become one of the most challenging issues in cucumber cultivation, aggravated by factors including long-term monocropping, declining soil fertility, and microbial community imbalances (Lian et al., 2023). In response, biological control strategies have gained increasing attention as sustainable and environmentally safe approaches to managing soil-borne diseases (Naranjo et al., 2015). Microbial-based biocontrol not only mitigates environmental pollution caused by excessive fertilizer and pesticide use but also improves soil health and reduces the recurrence of crop diseases (Nadarajah and Abdul Rahman, 2023).

Natural soil microorganisms, including bacteria, fungi, and protozoa, can exert beneficial, pathogenic, or neutral effects on host plants (Ortíz-Castro et al., 2009). Biocontrol agents derived from microorganisms can inhibit plant pathogens through multiple mechanisms, such as niche competition, nutrient rivalry, secretion of antibiotics and volatile compounds, lysozyme production, and induction of plant resistance (Marilley et al., 1998; Sun et al., 2022). When plants are attacked by fungal pathogens, biocontrol agents can help establish a “defense barrier” to combat infections (Trivedi et al., 2022; Hibbing et al., 2010; René et al., 2020). Plant rhizobacteria are an important group of soil microorganisms that play significant roles in promoting plant growth and enhancing disease resistance (René et al., 2020; Wang et al., 2021), thus emerging as a potential approach for controlling cucumber wilt.

*Bacillus velezensis*, as an emerging biocontrol microbial agent, has attracted considerable attention for its roles in plant disease management and plant growth promotion (Li et al., 2022). Numerous *Bacillus velezensis* strains, including *B. subtilis* (Xu et al., 2022), *Bacillus velezensis* (Luo et al., 2019), and *B. amyloliquefaciens* (Wu et al., 2015), have demonstrated broad-spectrum antagonism against phytopathogens through the synthesis of antifungal metabolites and niche competition (Keshmirshekan et al., 2024). Notably, *Bacillus velezensis* inhibits pathogens through multiple synergistic mechanisms, such as producing antifungal substances (e.g., lipopeptides, polyketides, and bacteriocins) via distinct metabolic pathways and inducing systemic resistance in host plants (Zhang et al., 2020; Xie et al., 2021). For instance, *Bacillus velezensis* strain YB15 secretes β-glucanase to suppress pathogenic fungi (Xu et al., 2014); strain VJH504 promotes plant growth by producing siderophores and indole-3-acetic acid (IAA) (Yang et al., 2023) and strain SH-1471 induces plant defense-related substances including pathogenesis-related (PR) proteins, secondary metabolites, and cyclic lipopeptides to limit pathogen invasion (Shen et al., 2023; Lau et al., 2020; Stoll et al., 2021). Other strains can induce systemic resistance by enhancing the activity of defense enzymes like superoxide dismutase (SOD), peroxidase (POD), phenylalanine ammonia lyase (PAL), polyphenol oxidase (PPO), and Catalase (CAT) (Zhai et al., 2023; Ha-Tran et al., 2021; Bhattacharyya et al., 2020). Furthermore, *Bacillus velezensis* has also been shown to promote plant growth through mechanisms such as phosphorus solubilization, nitrogen fixation, secretion of plant growth hormones, and enhancement of plant nutrient uptake. For example, *Bacillus velezensis* strain HNU24 facilitates tomato root growth (Cao et al., 2022); strain BAC03 exerts growth-promoting effects on crops such as cucumber, potato, and tomato (Meng et al., 2016); and strain CE100 inhibits pathogens including *Botrytis cinerea* and *Fusarium oxysporum* while producing IAA to promote plant growth (Moon et al., 2021). Thus, *Bacillus velezensis* has garnered widespread attention as a highly effective biocontrol agent in sustainable agriculture (Zhou et al., 2023).

In this study, we reported *Bacillus velezensis* LJ-19, which exerts dual functions: biocontrol against *Fusarium* wilt via a contact-dependent mechanism and possesses growth promotion in cucumber. These findings provide a sustainable approach for cucumber cultivation and pathogen control.

## 2 Materials and methods

### 2.1 Isolation of cucumber rhizosphere bacteria

Rhizosphere bacteria were isolated from soil samples collected from the root zone of cucumber plants following a modified protocol based on Li et al. (2020). Soil samples were randomly obtained from a cucumber greenhouse located on Donggan Road Street, Muye District, Xinxiang City, Henan Province, China (GPS coordinates: N35.3296, E113.9063). Four grams of each soil sample were mixed with 36 mL of sterile water and shaken for 20 minutes. The resulting rhizosphere bacterial suspension was serially diluted to 10^-6^. A 100 µL aliquot of the diluted suspension was spread onto Luria-Bertani (LB) agar plates and incubated for 16–18 hours. Colonies exhibiting distinct morphological features were selected and subcultured in fresh LB medium for 1–3 days. After three rounds of purification, the isolated colonies were screened for antagonistic activity against the fungal pathogen. The *Fusarium oxysporum* f. sp. *cucumerinum* used in this study was provided and preserved by the Henan Province Engineering Research Center of Biological Pesticide & Fertilizer Development and Synergistic Application.

### 2.2 Screening of cucumber rhizosphere bacteria antagonistic to *Fusarium oxysporum* f. sp. *cucumerinum*

The screening for rhizosphere bacteria with antagonistic activity against *Fusarium oxysporum* f. sp. *cucumerinum* was performed according to a previously described method (Wang et al., 2021), with minor modifications. A 5 mm mycelial disc of a candidate cucumber rhizosphere bacterial isolate and a 5 mm mycelial disc were placed on opposite sides of a potato dextrose agar (PDA) plate in 90 mm Petri dishes, with a distance of 50 mm between them. The plates were incubated at 25 °C for 7 days. A control plate was prepared with only a disc. The antagonistic effect was assessed by measuring the inhibition of hyphal growth; the diameter of the colony was used as a statistical indicator to quantify the level of antagonism. Each experiment was repeated three times.

### 2.3 Identification of cucumber rhizosphere bacterial strain LJ-19

To characterize the antagonistic strain LJ-19, its morphological and genetic features were examined following a previously described method (Ma et al., 2023), with minor modifications. Briefly, strain LJ-19 was streaked onto LB agar plates and incubated at 28 °C for 48 hours. Colony morphology, including edge, size, and color, was recorded. Gram staining was performed using a commercial kit (Beijing Solaibao Technology Co., Ltd.) and observed under an oil immersion microscope at 100× magnification. For molecular identification, genomic DNA was extracted from strain LJ-19 using a bacterial genomic DNA extraction kit (Beijing ComWin Biotech Co., Ltd.). The 16S rRNA gene was amplified by PCR with universal primers 27F:5’- AGAGTTTGATCATGGCTCAG-3’ and 1492R: 5’- TACGGCTA CCTTGTTACGA-3’). The 25 μL PCR reaction mixture contained 2 μL of DNA template, 1 μL of each primer, 8.5 μL of ddH_2_O, and 12.5 μL of 2× Taq plus Master Mix. The amplified product was purified and sequenced by Sangon Biotech (Shanghai) Co., Ltd. The resulting sequence was analyzed using the BLAST algorithm on the NCBI database, and a phylogenetic tree was constructed with MEGA 6.0 software (Wang et al., 2024). The resulting sequence was analyzed using the BLAST algorithm on the NCBI database, and a phylogenetic tree was constructed with MEGA 6.0 software.

### 2.4 Assessment of nitrogen fixation, phosphorus solubilization, and potassium solubilization by strain LJ-19

The nitrogen fixation, phosphorus solubilization, and potassium solubilization capabilities of strain LJ-19 were evaluated according to previously described methods (Zhai et al., 2023), with slight modifications. The strain was inoculated onto nitrogen-free Ashby medium, Pikovskaya (PVK) medium, and potassium feldspar (PF) solid medium, followed by incubation at 30 °C for 5 days. The formation of transparent zones around the colonies was examined as an indicator of nitrogen fixation, phosphorus solubilization, and potassium solubilization activities, respectively.

### 2.5 IAA production assay

To quantify IAA production, strain LJ-19 was initially cultured in liquid LB medium at 30 °C for 48 hours. A bacterial suspension with a concentration of 10^7^–10^8^ CFU/mL was then transferred into a nutrient broth supplemented with 100 μg/mL tryptophan and incubated in the dark at 30 °C for 72 hours. After incubation, the culture was centrifuged at 12,000 rpm for 5 minutes to collect the supernatant. Then, 3 mL of the supernatant was mixed with an equal volume of Salkowski reagent and incubated in darkness at 30 °C for 30 minutes. The development of a pink color indicated IAA production. A medium control without bacterial inoculation was included. The absorbance of the solution was measured at 540 nm using a spectrophotometer to confirm IAA synthesis (Zhai et al., 2023).

### 2.6 Siderophore production assay

Siderophore production was qualitatively assessed using the chrome azurol S (CAS) agar plate method as described by Zhai et al. (2023). Strain LJ-19 was inoculated onto CAS agar plates and incubated at 28 °C for 7 days. Siderophores secreted by the strain chelate iron from the iron-CAS complex in the medium, resulting in the formation of an orange halo around the bacterial colony. The presence of an orange halo around the colony of LJ-19 was considered a positive indicator of siderophore production.

### 2.7 Effect of strain LJ-19 on the induction of defense enzymes in cucumber

Uniformly grown cucumber seedlings (Zhongnong No. 8) were selected as experimental materials. The seedlings were cultivated in a greenhouse under the following conditions: temperature maintained between 24 °C and 28 °C, a photoperiod of 16 hours light/8 hours dark, and relative humidity ranging from 64% to 83%. During the two-leaf stage, the seedlings were transplanted into plastic pots, with 10 seedlings per pot. Four treatment groups were established as follows: the first group was treated with sterile water(control); the second group was treated with a bacterial suspension of strain LJ-19 (1 × 10^8^ CFU/mL); the third group was treated with a suspension of the cucumber wilt pathogen (1 × 10^6^ spores/mL); the fourth group was treated with both the LJ-19 bacterial suspension (1 × 10^8^ CFU/mL) and pathogen suspension (1 × 10^6^ spores/mL). After 96 hours of treatment, root samples were collected from the cucumber seedlings. A total of 0.1 g of root tissue was homogenized in 1 mL extraction buffer of the specific assay kit (Suzhou Greis Biotechnology Co., Ltd., China) using a pre-cooled mortar and pestle. The homogenate was kept at 4 °C during processing and then centrifuged at 10,000 rpm for 10 minutes at 4 °C. The resulting supernatant was collected, and its absorbance was measured at 240 nm, 290 nm, 470 nm, 560 nm, and 495 nm to determine the activities of CAT, PAL, POD, SOD, and PPO, respectively. Each treatment was repeated three times (Wang et al., 2020b).

### 2.8 Quantification of gene expression using RT-qPCR

Quantitative real-time PCR (RT-qPCR) was performed to analyze the transcript levels of defense-related genes in cucumber plants treated with strain LJ-19 or sterile water (control) following inoculation with *Fusarium oxysporum* f. sp. *cucumerinum*. The root samples from cucumber plants were collected at 72 hours post-inoculation according to the method described by Pu et al. (2014). For each sample, 0.1 g of tissue was homogenized in 1 mL of TRI Gene Reagent (purchased from Nanjing Vazyme Biotech Co., Ltd., China) using a pre-cooled mortar and pestle. The expression of the following genes was examined: *Actin* (internal reference), *PR1*, *PR3*, *LOX1*, *CTR1*, and *PAL1*. Gene-specific primers were designed using Primer Premier 5.0 based on the cDNA sequences of each target gene (**Supplementary Table S1**). RT-qPCR was carried out using an ABI 7500 Real-Time PCR System. Each 15 µL reaction mixture contained: 7.5 μL of 2× TransStart™ Green qPCR SuperMix, 0.5 μL of passive reference dye II, 0.5 μL of each forward and reverse primer, 6 μL of ddH_2_O, and 0.5 μL of cDNA template. The amplification protocol consisted of an initial denaturation at 95 °C for 2 minutes, followed by 40 cycles of 95 °C for 15 seconds, 59 °C for 30 seconds, and 72 °C for 35 seconds. The Actin gene was used as an endogenous control to normalize cDNA levels across samples. Relative gene expression was calculated using the 2^-ΔΔCt^ method. Each treatment included fifteen seedlings, and data are presented as the mean ± standard deviation from three independent biological replicates (Pu et al., 2014).

### 2.9 Poison food technique

Strain LJ-19 was cultured in 250 mL of LB medium at 28 °C for 7 days with shaking at 180 r/min. After fermentation, the broth was centrifuged at 8000 × g for 10 minutes to collect the supernatant. The supernatant was then filter-sterilized using a 0.22 μm membrane and incorporated into PDA plates inoculated with *Fusarium oxysporum* f. sp. *cucumerinum* to assess antagonistic activity. The presence of a growth inhibition zone around the *Fusarium oxysporum* f. sp. *cucumerinum* inoculum indicated antagonistic effects attributable to the extracellular metabolites produced by strain LJ-19 (Dong et al., 2023).

### 2.10 Effect of strain LJ-19 on *Fusarium oxysporum* f. sp. *cucumerinum* spore germination

*Fusarium oxysporum* f. sp. *cucumerinum* was cultured on potato dextrose agar (PDA) medium, and spores were harvested to prepare a spore suspension. Three treatment groups were established as follows: the first group served as a control, LB medium without bacteria was mixed with the *Fusarium oxysporum* f. sp. *cucumerinum* spore suspension in a 1:1 ratio; the second group mixed the liquid culture of strain LJ-19 was mixed with the *Fusarium oxysporum* f. sp. *cucumerinum* spore suspension in a 1:1 ratio; and the third group mixed the filter-sterilized extracellular metabolite filtrate of strain LJ-19 was mixed with the *Fusarium oxysporum* f. sp. *cucumerinum* spore suspension in a 1:1 ratio. The mixtures were placed on concave slides and incubated at 28 °C. After 48 hours, spore germination was observed under a MOTIC SK160 microscope (MOTIC CHINA GROUP CO., LTD.). A conidium was considered germinated when the germ tube length exceeded half of the conidium length. Each treatment was repeated three times (Li et al., 2015).

### 2.11 Effect of strain LJ-19 on seed germination and seedling growth of cucumber

Cucumber seeds were surface-disinfected to remove external contaminants. The seeds were rinsed with distilled water, soaked in 75% alcohol for 3 min, and then immersed in a 5% sodium hypochlorite solution for 30 seconds, followed by three washes with sterile distilled water. Two main treatment groups were established for the germination assay: First group: thirty seeds were soaked in sterile water for 6 hours. Half of these were then transferred to a bacterial suspension of strain LJ-19 (1 × 10^7^ CFU/mL) for 1 hour. Second group: thirty seeds were soaked in the LJ-19 bacterial suspension (1 × 10^7^ CFU/mL) for 6 hours. Half were then treated with a *Fusarium oxysporum* f. sp. *cucumerinum* spore suspension (1 × 10^6^ spores/mL) for 20 minutes, while the other half were soaked in sterile water for 20 minutes. Germination was monitored daily. The following parameters were calculated: germination rate (percentage of normal seedlings), germination index (GI), and seedling vigor index (SVI). GI was calculated as the cumulative number of germinated seeds divided by the number of days after sowing, assessed over 7 days. SVI was determined by multiplying GI by seedling fresh weight. After 7 days, seedlings at the cotyledon stage were transplanted into pots. The experimental group received irrigation with the LJ-19 bacterial suspension (1 × 10^7^ CFU/mL), while the control group was irrigated with sterile water. Plants were grown in a greenhouse under the following conditions: temperature 24–28 °C, 16-h light/8-h dark photoperiod, and relative humidity 64–83%, with 10 seedlings per pot. Each treatment was replicated three times. After 45 days, plant growth parameters were recorded. Seedlings were carefully removed from the pots, and plant height, root length, fresh weight, biomass, and maximum leaf area were measured to evaluate the growth-promoting effects of strain LJ-19 (Yang et al., 2024).

### 2.12 Effect of strain LJ-19 on cucumber disease prevention

Strain LJ-19 was cultured in LB medium at 30 °C for 24 hours with shaking at 180 r/min, and the bacterial concentration was adjusted to 10^8^ CFU/mL based on OD_600_ measurement. *Fusarium oxysporum* f. sp. *cucumerinum* was grown on PDA medium at 28 °C for 5 days. Then, ten 5-mm mycelial plugs were excised and transferred into 100 mL of potato dextrose broth (PDB), followed by incubation in a shaker at 28 °C and 180 r/min for 3 days. The resulting culture was filtered through four layers of sterile gauze, and the spore concentration was adjusted to 10^5^ spores/mL. Uniform cucumber seeds were surface-sterilized by soaking in 5% sodium hypochlorite for 30 seconds and 75% ethanol for 3 minutes, then rinsed three times with sterile water. The seeds were placed in sterile Petri dishes and transferred to seedling trays for growth in a greenhouse at 25 °C under a 16-h light/8-h dark photoperiod until the two-leaf stage was reached. Each pot of cucumber seedlings was subjected to one of the following treatments: (1) Soaking with 15 mL of *Fusarium oxysporum* f. sp. *cucumerinum* spore suspension; (2) Soaking with 15 mL of LJ-19 bacterial suspension followed, after 24 hours, by soaking with 15 mL of *Fusarium oxysporum* f. sp. *cucumerinum* spore suspension. After treatment, the seedlings were transplanted into plastic pots. Each treatment consisted of 12 cucumber seedlings and was repeated three times. The disease index of plants inoculated with *Fusarium oxysporum* f. sp. *cucumerinum*, with or without LJ-19 pretreatment, was recorded 25 days after inoculation (Xu et al., 2022).

### 2.13 Statistical analysis

All experimental data were processed using Origin 2019 software. Statistical significance of differences was assessed through analysis of variance (ANOVA) performed with SPSS (version 19.0 for Windows, USA). Pairwise comparisons were carried out using t-tests and one-way ANOVA implemented in GraphPad Prism software. Data are presented as mean ± standard deviation (SD). The following symbols were used to denote significance levels: *P* < 0.05. NS, not significant.

## 3 Results

### 3.1 Cucumber rhizobacterium LJ-19 inhibits hyphal growth of *Fusarium oxysporum* f. sp. *cucumerinum*

**Figure 1:**
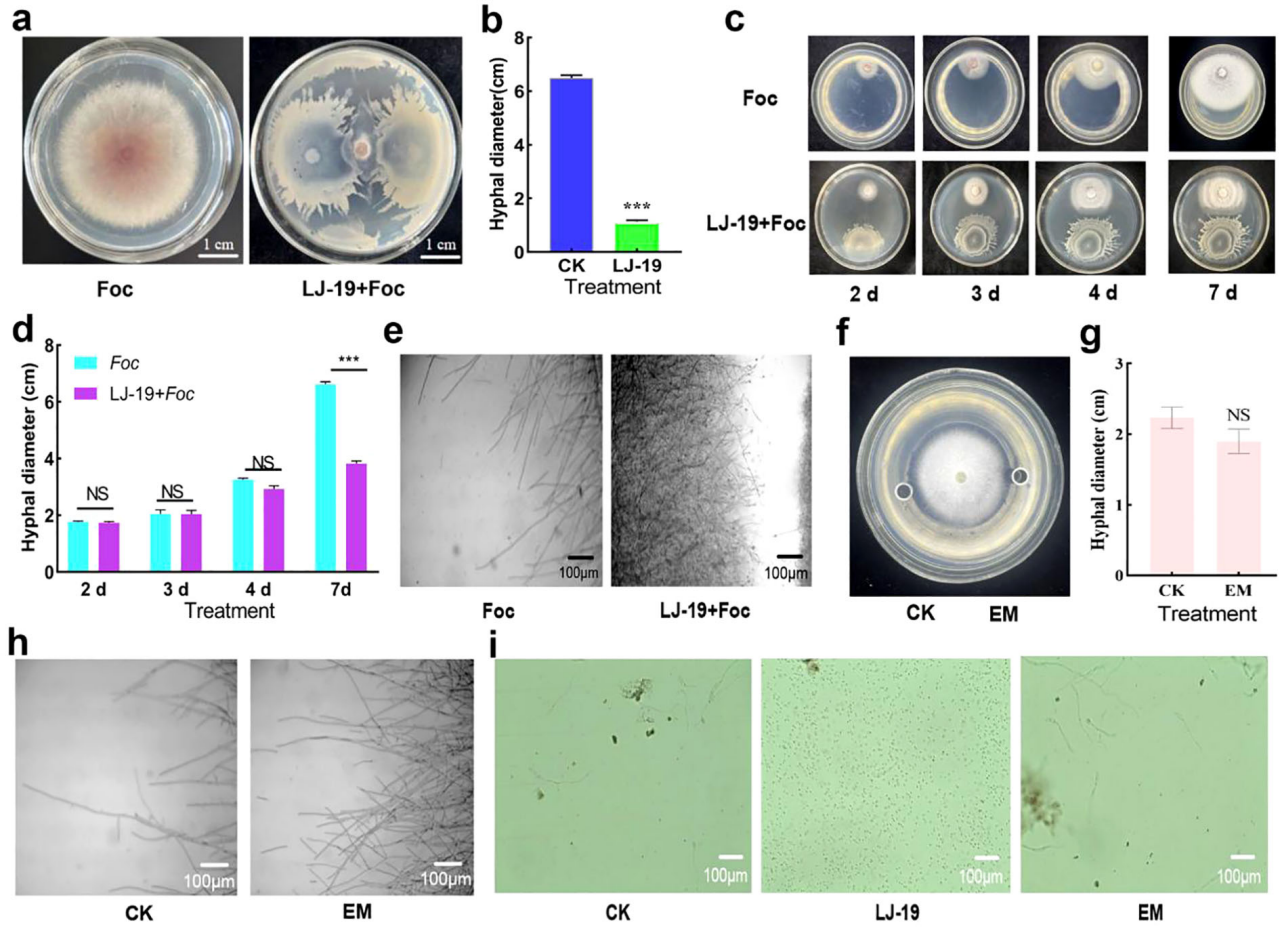
Contact-dependent antagonistic activity of *Bacillus velezensis* LJ-19 against *Fusarium oxysporum* f sp. *cucumerinum*. **(a)** Inhibition of *Fusarium oxysporum* f sp. *cucumerinum* growth by LJ-19. **(b)** Effect of LJ-19 treatment on *Fusarium oxysporum* f sp. *cucumerinum* colony diameter. **(c)** Hyphal growth of *Fusarium oxysporum* f sp. *Cucumerinum* before contact with LJ-19. **(d)** Changes in hyphal diameter of *Fusarium oxysporum* f sp. *cucumerinum* before contact with LJ-19. **(e)** Microscopic morphology of *Fusarium oxysporum* f sp. *cucumerinum* hyphae after contact with LJ-19. **(f)** Antagonistic inhibition of extracellular metabolites hyphae. **(g)** The diameter of *Fusarium oxysporum* f sp. *cucumerinum* after LJ-19 extracellular metabolites treatment. **(h)** Microscopic morphology of *Fusarium oxysporum* f sp. *cucumerinum* hyphae grown with extracellular metabolites of LJ-19. **(i)** The effects of strain LJ-19 and its extracellular metabolites on the development of *Fusarium oxysporum* f sp. *cucumerinum* spores. Foc: *Fusarium oxysporum* f sp. *cucumerinum.* Error bars represent standard deviation (SD) of the mean (n = 3). Asterisks indicate significant differences: *** *p* < 0.001; NS, Non-significant.

In this study, 156 bacterial strains were isolated from the cucumber rhizosphere and screened for antagonistic activity against *Fusarium oxysporum* f. sp. *cucumerinum*. Among these, strain LJ-19 exhibited the strongest antifungal activity (**Figures 1a b**, **Supplementary Table S2**), with an inhibition rate of 83.59% (**Figure 1c**). To determine whether inhibition required physical contact, the distance between LJ-19 and *Fusarium oxysporum* f. sp. *cucumerinum* was varied. When not in direct contact, the hyphal diameter of *Fusarium oxysporum* f. sp. *Cucumerinum* showed no significant difference compared to the control. In contrast, hyphal growth was completely inhibited upon direct contact with LJ-19 (**Figures 1d, e**). Furthermore, microscopic observation revealed that upon contact with LJ-19, *Fusarium oxysporum* f. sp. *cucumerinum* hyphae ceased polarized growth, exhibited increased branching, and underwent severe structural alterations. Compared to the control, most hyphae lost their original morphology, forming irregular clusters with apparent surface shrinkage and deformation (**Figure 1f**). Together, these results indicate that strain LJ-19 inhibits *Fusarium oxysporum* f. sp. *cucumerinum* hyphal growth through direct contact, resulting in morphological disruption and impaired fungal development.

### 3.2 Effect of cucumber rhizobacterium LJ-19 on the severity of cucumber wilt disease

**Figure 2:**
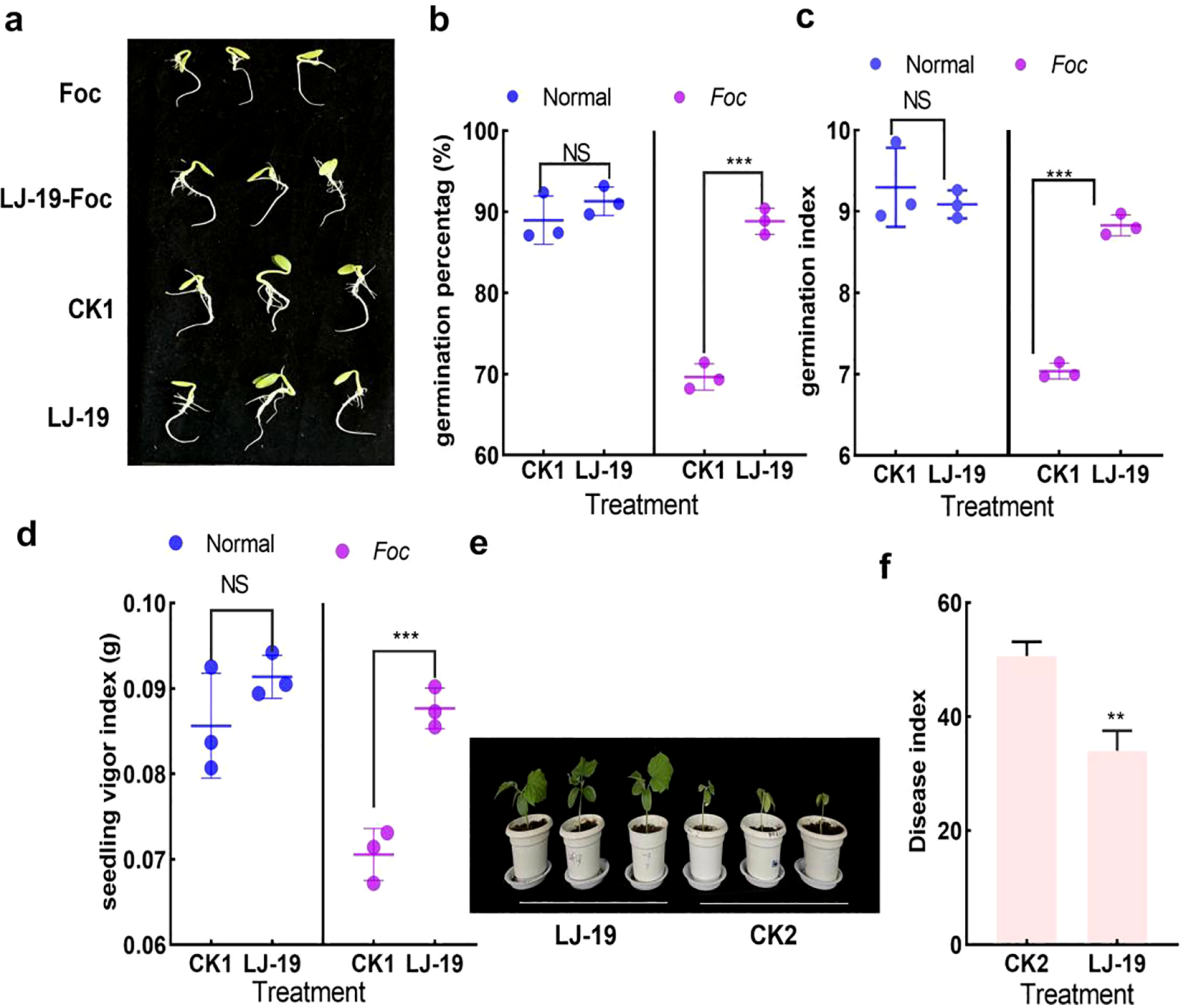
Effect of *Bacillus velezensis* strain LJ-19 on seed germination and disease index of cucumber wilt disease. **(a)** Seed germination rate; **(b)** Germination potential; **(c)** Germination index; **(d)** Seedling vigor; **(e)** Phenotype of cucumber seedlings; **(f)** Disease index of cucumber wilt. CK1, untreated control; CK2, inoculated with *Fusarium oxysporum* f sp. *cucumerinum* only. Values represent mean ± SD (n = 3). Significance levels: ** *P* < 0.01; *** *P* < 0.001; NS, Non-significant.

Although the antagonistic effect of cucumber rhizobacterium LJ-19 against *Fusarium oxysporum* f. sp. *cucumerinum* was established, its ability to mitigate *Fusarium oxysporum* f. sp. *Cucumerinum* induced damage in cucumber plants warranted further investigation. The germination rate of cucumber seeds was evaluated following treatment with LJ-19 and *Fusarium oxysporum* f. sp. *cucumerinum*, both individually and in combination. Under normal conditions, seed germination was comparable between LJ-19-treated and untreated groups (**Figure 2a**). However, under *Fusarium oxysporum* f. sp. *cucumerinum* infection, seeds treated with LJ-19 showed significantly higher germination percentage, germination index, and seedling vigor index compared to the infected control. In contrast, *Fusarium oxysporum* f. sp. *cucumerinum* infection alone resulted in markedly reduced values across these indices relative to the sterile water control (**Figures 2b–d**). Additionally, cucumber seedlings treated with LJ-19 exhibited a substantially lower disease incidence, with a disease index of 34, compared to the control value of 50.68 (**Figures 2e, f**). These findings indicate that strain LJ-19 effectively alleviates *Fusarium oxysporum* f. sp. *cucumerinum* induced damage and delays the onset of cucumber wilt disease.

### 3.3 Morphological and molecular identification of cucumber rhizobacterium LJ-19

**Figure 3:**
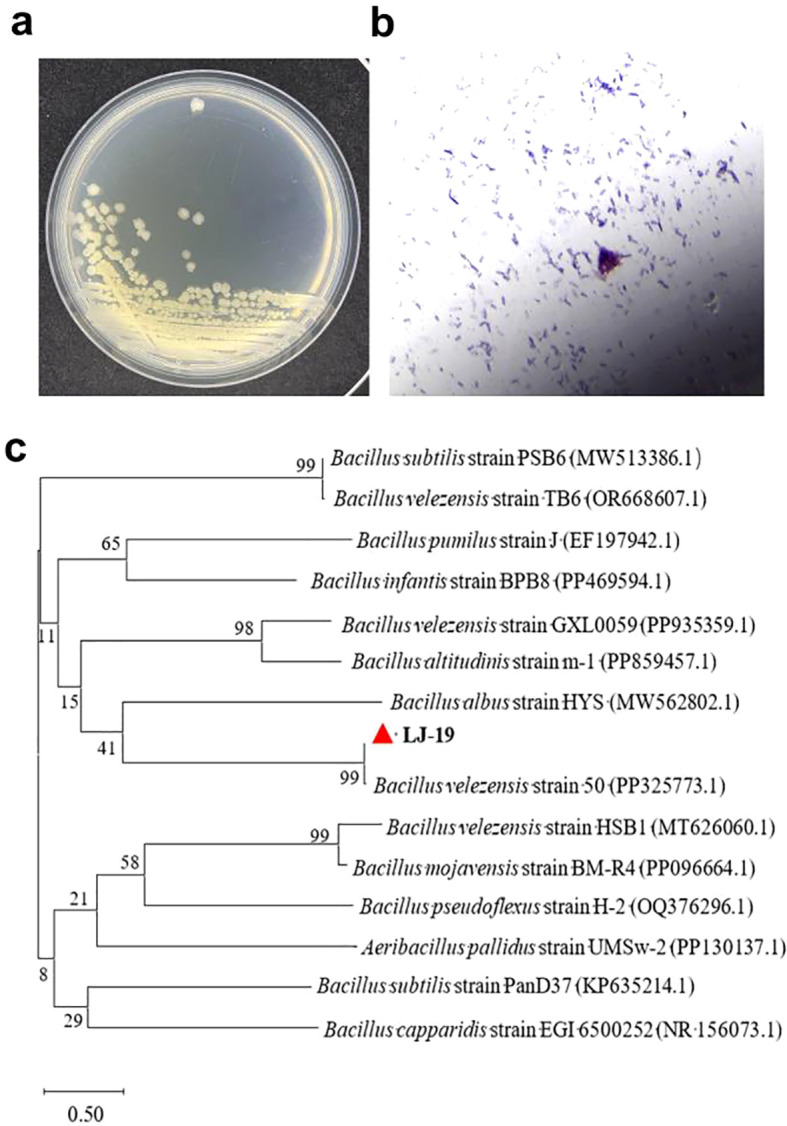
Characterize of the cucumber rhizobacterium LJ-19. **(a, b)** Morphological, physiological, and biochemical characterization of strain LJ-19. **(c)** Phylogenetic tree of strain LJ-19 based on 16S rDNA gene sequence.

To characterize the cucumber rhizobacterium LJ-19, both morphological and molecular biological identification were carried out. Colonies of strain LJ-19 appeared round, opaque, pale yellow, with irregular margins, rough surfaces, and raised profiles. Physiological and biochemical assays confirmed that the strain is Gram-positive and rod-shaped (**Figures 3a, b**, **Supplementary Figure S1**, **Supplementary Table S3**). The 16S rDNA gene was amplified from strain LJ-19 and sequenced. Phylogenetic analysis based on the 16S rDNA sequence revealed that LJ-19 shares high homology with members of the *Bacillus velezensis* group and clusters within this species in the phylogenetic tree (**Figure 3c**). Combined results from colonial and cellular morphology, physiological and biochemical characteristics, and molecular identification confirm that strain LJ-19 belongs to *Bacillus velezensis*.

### 3.4 Plant growth-promoting activity of strain LJ-19 *in vitro*

**Figure 4:**
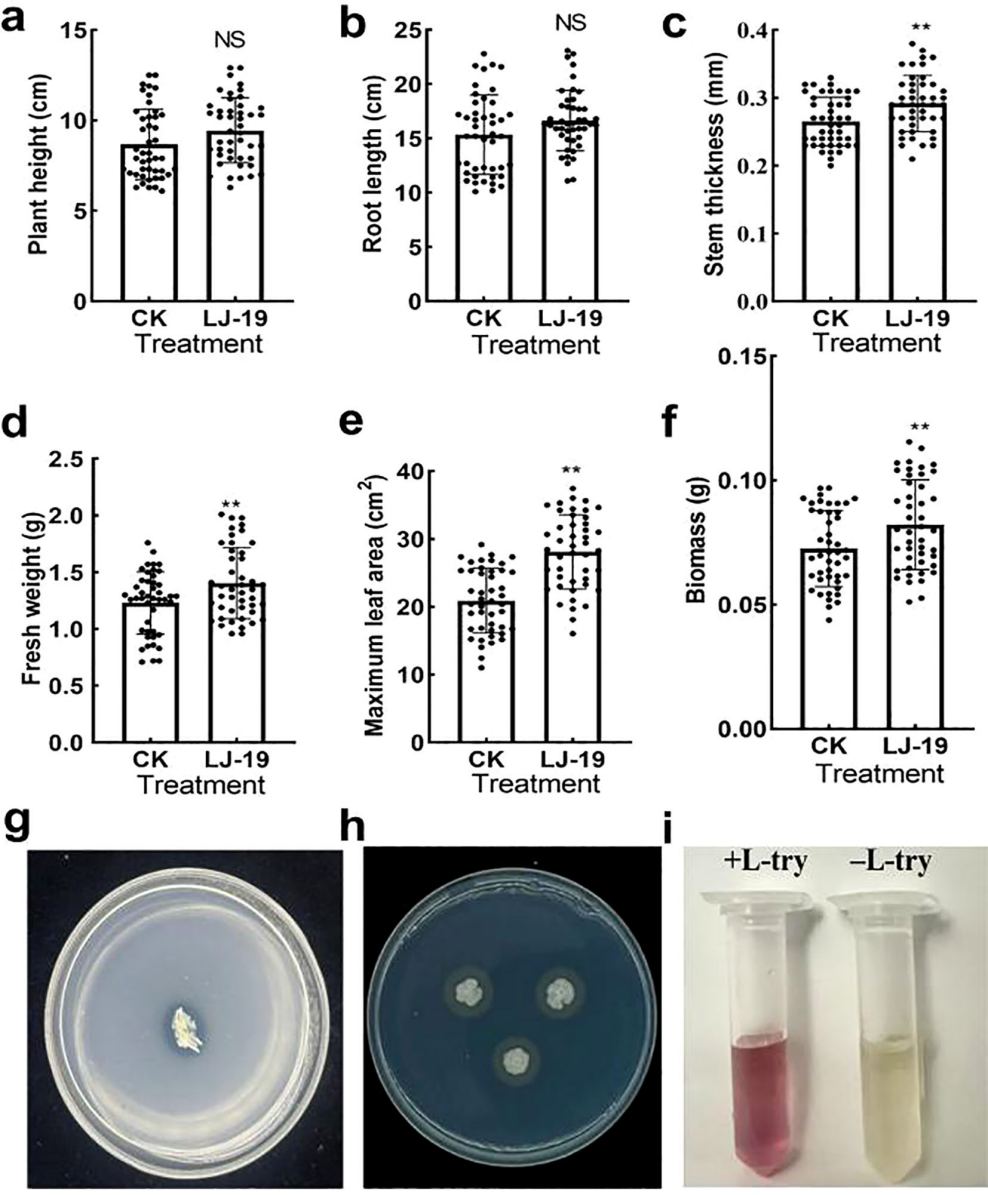
Effect of LJ-19 on cucumber growth. **(a-f)** Growth promotion of cucumber by strain LJ-19; **(g)** Phosphate-solubilizing activity; **(h)** Siderophore production; **(i)** IAA producing activities. Error bars represent standard deviation (SD) of the mean (n = 45). Significance levels: **, *P* < 0.01; NS, Non-significant.

As a cucumber rhizosphere microorganism, strain LJ-19 not only exhibited antagonistic activity against *Fusarium oxysporum* f. sp. *cucumerinum* but also demonstrated potential for enhancing plant growth. To evaluate its growth-promoting effects, key parameters of cucumber plants, including stem thickness, root length, maximum leaf area, fresh weight, and dry weight, were measured following treatment with LJ-19. Compared to the control, LJ-19 treatment led to significant increases in stem thickness (15.44 ± 0.04%), fresh weight (13.82 ± 0.3%), maximum leaf area (37.75 ± 6.31%), and biomass (13.22 ± 0.02%) (**Figures 4a–f**), indicating pronounced growth promotion. The plant growth-promoting potential of LJ-19 was further assessed *in vitro*. When cultured on phosphorous-solubilizing medium for three days, the strain formed a distinct dissolution halo (**Figure 4g**), confirming its ability to solubilize inorganic phosphorus. No halos were observed on nitrogen-free Ashby, Pikovskaya (PVK), or potassium feldspar (PF) media, supporting the specificity of its phosphorus-solubilizing trait. Additionally, the formation of an orange halo around LJ-19 colonies on chrome azurol S (CAS) medium indicated siderophore production (**Figure 4h**), which facilitates iron acquisition in plants. Moreover, the culture supernatant supplemented with L-tryptophan developed a deeper red color compared to the unsupplemented control, verifying the synthesis of IAA (**Figure 4i**). These findings demonstrate that *Bacillus velezensis* LJ-19 is capable of producing siderophores, solubilizing phosphorus, and synthesizing IAA *in vitro*, which may collectively contribute to its plant growth-promoting effects in cucumber.

### 3.5 Detection of defense enzyme activities induced by strain LJ-19 in cucumber in response to *Fusarium* wilt

**Figure 5:**
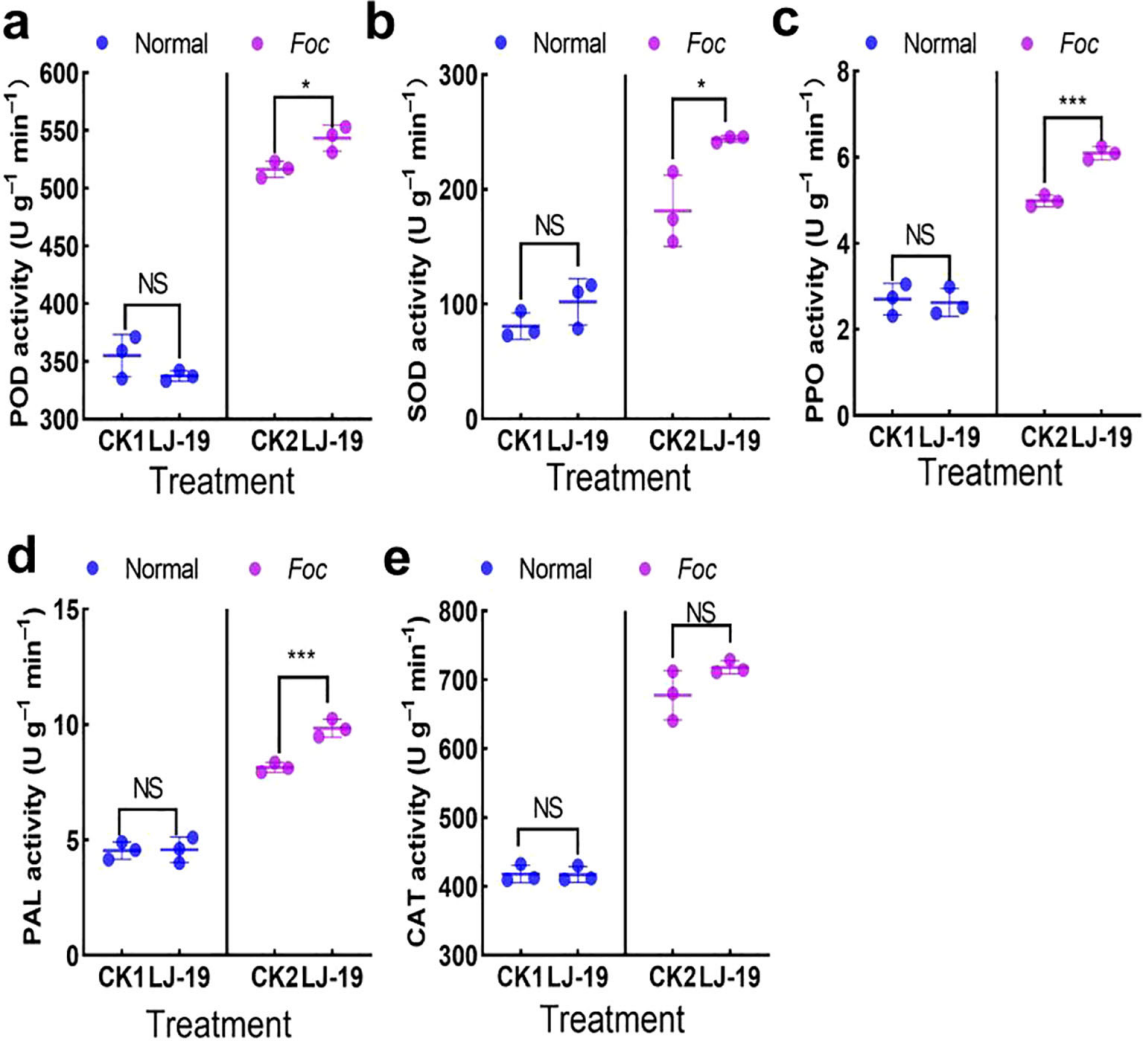
Effects of strain LJ-19 on the activities of defense enzymes in cucumber under *Fusarium* wilt stress. **(a)** Peroxidase (POD); **(b)** Superoxide dismutase (SOD); **(c)** Polyphenol oxidase (PPO); **(d)** Phenylalanine ammonia-lyase (PAL); **(e)** Catalase (CAT). CK1, untreated control; CK2, inoculated with *Fusarium oxysporum* f sp. *cucumerinum* only. Error bars represent mean ± SD (n = 3). Significance levels: * *P* < 0.05; *** *P* < 0.001; NS, Non-significant.

To determine whether strain LJ-19 enhances the defense response in cucumber, the activities of several defense-related enzymes were measured. The results showed that under *Fusarium* wilt stress, treatment with strain LJ-19 significantly increased the activities of several defense enzymes in cucumber roots compared to the control (CK) group: POD by 5.22% (**Figure 5a**), SOD by 34.59% (**Figure 5b**), and PPO by 22.04% (**Figure 5c**). Furthermore, LJ-19 treatment markedly enhanced PAL activity both under pathogen stress and in the absence of *Fusarium oxysporum* f. sp. *cucumerinum*, with a stronger induction effect than that caused by *Fusarium* wilt infection alone (**Figure 5d**). In contrast, no significant effect on CAT activity was observed (**Figure 5e**). These results indicate that strain LJ-19 induces the activity of defense enzymes POD, SOD, PPO, and PAL, thereby enhancing cucumber resistance to *Fusarium oxysporum* f. sp. *cucumerinum*.

### 3.6 Expression pattern analysis of defense-related genes

**Figure 6:**
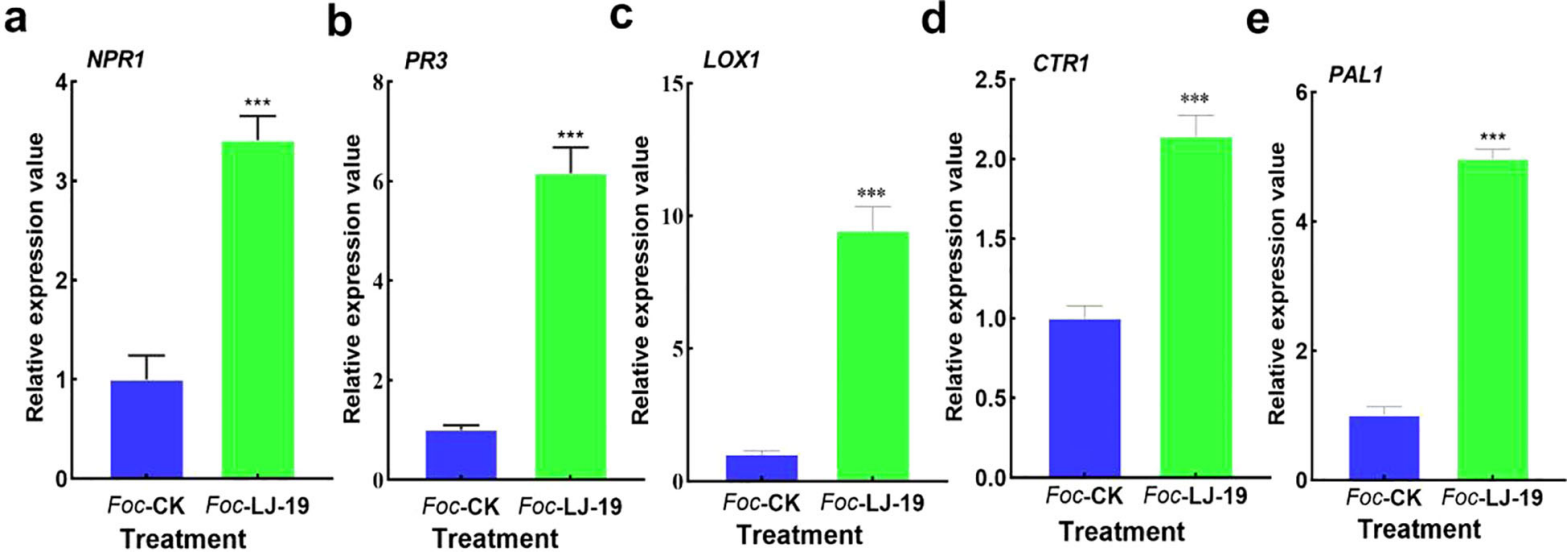
Expression levels of defense-related genes in cucumber roots. **(a)***NPR1*; **(b)***PR3*; **(c)***LOX1*; **(d)***CTR1*; **(e)***PAL1*. Data are presented as mean ± SD (n = 3). Asterisks indicate statistically significant differences, *** *P* < 0.001.

The expression dynamics of defense-related genes in cucumber roots treated with strain LJ-19 and inoculated with *Fusarium oxysporum* f. sp. *cucumerinum* were analyzed using quantitative real-time PCR (RT-qPCR). Defense-related genes play essential roles in plant disease resistance, with many being induced by beneficial rhizosphere microorganisms. Our results showed transcript levels of several key defense-related genes were significantly up-regulated 72 hours after treatment with strain LJ-19. Specifically, NPR1—a marker gene in the salicylic acid (SA) pathway—was up-regulated 3.14-fold (**Figure 6a**). PR3 expression increased by 6.16-fold compared to the *Fusarium oxysporum* f. sp. *cucumerinum* only treatment group (**Figure 6b**), suggesting its involvement in the induced defense response. LOX1, which is associated with lipid metabolism regulation, showed a 9.43-fold increase (**Figure 6c**). CTR1, a regulator of the ethylene signaling pathway, was up-regulated 2.14-fold (**Figure 6d**), while PAL1, critical for secondary metabolite synthesis, exhibited a 4.97-fold increase in expression (**Figure 6e**). Collectively, the up-regulation of *NPR1*, *PR3*, *LOX1*, *CTR1*, and *PAL1* indicates that strain LJ-19 activates multiple metabolic pathways linked to plant disease resistance, enhancing cucumber’s ability to combat *Fusarium oxysporum* f. sp. *cucumerinum* infection.

## 4 Discussion

The occurrence of plant soil-borne diseases is governed by complex interactions within the rhizosphere microbiome. Rhizosphere biodiversity plays a crucial role in modulating these interactions and influencing disease outcomes (Gao et al., 2021; Abbass et al., 2022). Rhizobacteria can reshape microbial community structure, enhance soil enzyme activities, and improve nutrient availability, thereby promoting plant growth and inducing systemic resistance against pathogens (Saeed et al., 2021; Santoyo et al., 2021). Among them, *Bacillus velezensis* has garnered widespread attention due to its numerous beneficial traits, including regulating the rhizosphere microbiome, increasing microbial diversity, enriching beneficial microorganisms, mobilizing nutrients, promoting plant growth, inhibiting pathogen infection, and activating plant immunity (Fazle Rabbee and Baek, 2020; Wang et al., 2020a; Liu et al., 2021; Wang et al., 2024). For instance, *Bacillus velezensis* strain B4–7 reduces the incidence of tobacco wilt disease and promotes plant growth (Meng et al., 2024); strain C16 inhibits Alternaria solani via lipopeptides and volatile organic compounds (Zhang et al., 2021a); and strain HN-2 induces antioxidant defense mechanisms in pepper to resist viral infection (Xuan et al., 2024). Currently, *Bacillus velezensis* has emerged as a crucial resource for the green control of crop diseases, as it can effectively inhibit the growth and pathogenicity of phytopathogenic fungi through multiple mechanisms. In this study, the rhizobacterial strain LJ-19 isolated from cucumber rhizosphere was identified as *Bacillus velezensis* (**Figure 3**). This strain exhibited significant antagonistic activity against *Fusarium oxysporum* f. sp. *cucumerinum* with an inhibition rate of 83.59% (**Figure 1c**), indicating strain L-19 holds potential for the development of biocontrol agents.

Traditional biocontrol *Bacillus velezensis* exhibit diverse mechanisms of action, with the most representative being their secreted antimicrobial metabolites—a process that does not rely on direct contact between biocontrol bacteria and pathogens (Deveau et al., 2018; Steffan et al., 2020; Zhou et al., 2022). For example, Phenazine-1-carboxamide secreted by *Pseudomonas chlororaphis* ZJU60 can directly target topoisomerase IV (Topo IV) of *Bacillus subtilis*, thereby inhibiting bacterial cell division (Zhou et al., 2025). *Bacillus velezensis* XT1 metabolized surfactin to inhibit the infection of *Botrytis cinerea* in tomatoes (Toral et al., 2018) and strain TP-1 enhanced grape resistance to gray mold by inducing the activities of plant defense enzymes PAL, PPO, and POD in grapes (Zou et al., 2023). *Corallococcus* sp. strain EGB controlled cucumber *Fusarium* wilt by migrating to the plant root and regulating the soil microbial community (Ye et al., 2020). In our study, LJ-19 treatment significantly enhanced the activities of PPO, POD, SOD, and PAL in cucumber plants under *Fusarium oxysporum* f. sp. *cucumerinum* challenge (**Figure 5**), indicating a robust biochemical defense response elicited by LJ-19. PPO and POD contribute to pathogen inhibition via quinone formation and lignin polymerization (Kumar and Ebel, 2016; Dos Santos-Costa et al., 2022), while SOD mitigoxidative stress by scavenging reactive oxygen species (Zhang et al., 2021b). PAL is essential for synthesizing phenylpropanoid-derived defense compounds (Cuéllar-Torres et al., 2022). Moreover, LJ-19 induced the expression of key defense-related genes (**Figure 6**). *NPR1*, a central regulator of salicylic acid-dependent immunity, was up-regulated, along with PR3, which encodes a pathogenesis-related protein involved in antifungal defense (Zavaliev and Dong, 2024) The increased expression of *LOX1* suggests activation of oxylipin signaling (Gousiadou and Kouskoumvekaki, 2018), while up-regulation of *CTR1* and *PAL1* reflects modulation of ethylene signaling and phenolic metabolism, respectively (Chen et al., 2022; Park et al., 2023). These results indicate that strain LJ-19 can simultaneously trigger the plant’s multi-pathway defense response, thereby enhancing the plant’s ability to resist infection by *Fusarium oxysporum* f. sp. *cucumerinum*.

In recent years, a contact-based antibacterial activity mode independent of antifungal metabolites have garnered increasing attention, which was achieved through intercellular contact between bacterial cells and fungal conidia, and provided a novel idea for plants to resist fungal infections (Lin et al., 2025). Studies have shown that beneficial bacteria can effectively defend against pathogenic fungal infections through type VI secretion system (T6SS/T4SS), contact-dependent growth inhibition (CDI) systems, and antibiotic-independent intercellular interactions (Nikolakakis et al., 2012; Klein et al., 2020; Shen et al., 2021; Wu et al., 2021). *Pseudomonas aeruginosa* PAO1 harbors multiple functional T6SS gene clusters and secretes a variety of antibacterial effector proteins (e.g., Tse1, Tse2, and Tse3), exerting a potent inhibitory effect on a wide range of gram-negative bacteria (Sana et al., 2016). *Lysobacter enzymogenes* OH11 inhibited the infection of *Xanthomonas citri* by secreting toxic effectors via the type IV secretion system (T4SS) (Shen et al., 2021). *Pseudomonas* strains 2P24 and FoE9 inhibited the growth of *Fusarium* in a contact-dependent manner mediated by T6SS (Lin et al., 2025). *Escherichia coli* EC93 possesses a functional CDI system, which can deliver the CdiA-CT toxin to neighboring bacteria through cell contact, inhibiting their growth (Nikolakakis et al., 2012). In our study, LJ-19 did not suppress hyphal development of *Fusarium oxysporum* f. sp. *cucumerinum* via extracellular compounds (**Figures 1f–i**). Instead, it significantly reduced germ tube elongation of the pathogen’s spores during direct coculture (**Figures 1a–e**), indicating that its antagonism primarily operates through contact-dependent mechanisms—rarely reported in *Bacillus velezensis*. This distinctive mode of action, differing from other strains of the species and diffusional antibiotics, has potential advantages in reducing the risk of drug resistance and accurately targeting pathogenic bacteria and also has great advantages in adaptability in microbial community competition and niche occupation, which provides a novel strategy for pathogen control. In addition, LJ-19 was demonstrated multiple plant growth-promoting traits (**Figures 4a–f**). It solubilized phosphorus, produced siderophores, and synthesized IAA (**Figures 4g–i**)—key mechanisms that enhance nutrient availability and stimulate plant development (Zhang et al., 2024; Martinez-Viveros et al., 2010; Zhou et al., 2021; Ma et al., 2023; Ta et al., 2024), which were consistent with other reported *Bacillus velezensis* strains that simultaneously promote growth, such as strain YXDHD1–7 in tomato against early blight (Li et al., 2024). These results indicate that strain LJ-19 possesses dual functions in controlling *Fusarium* wilt of cucumber and promoting cucumber growth.

## 5 Conclusions

**Figure 7:**
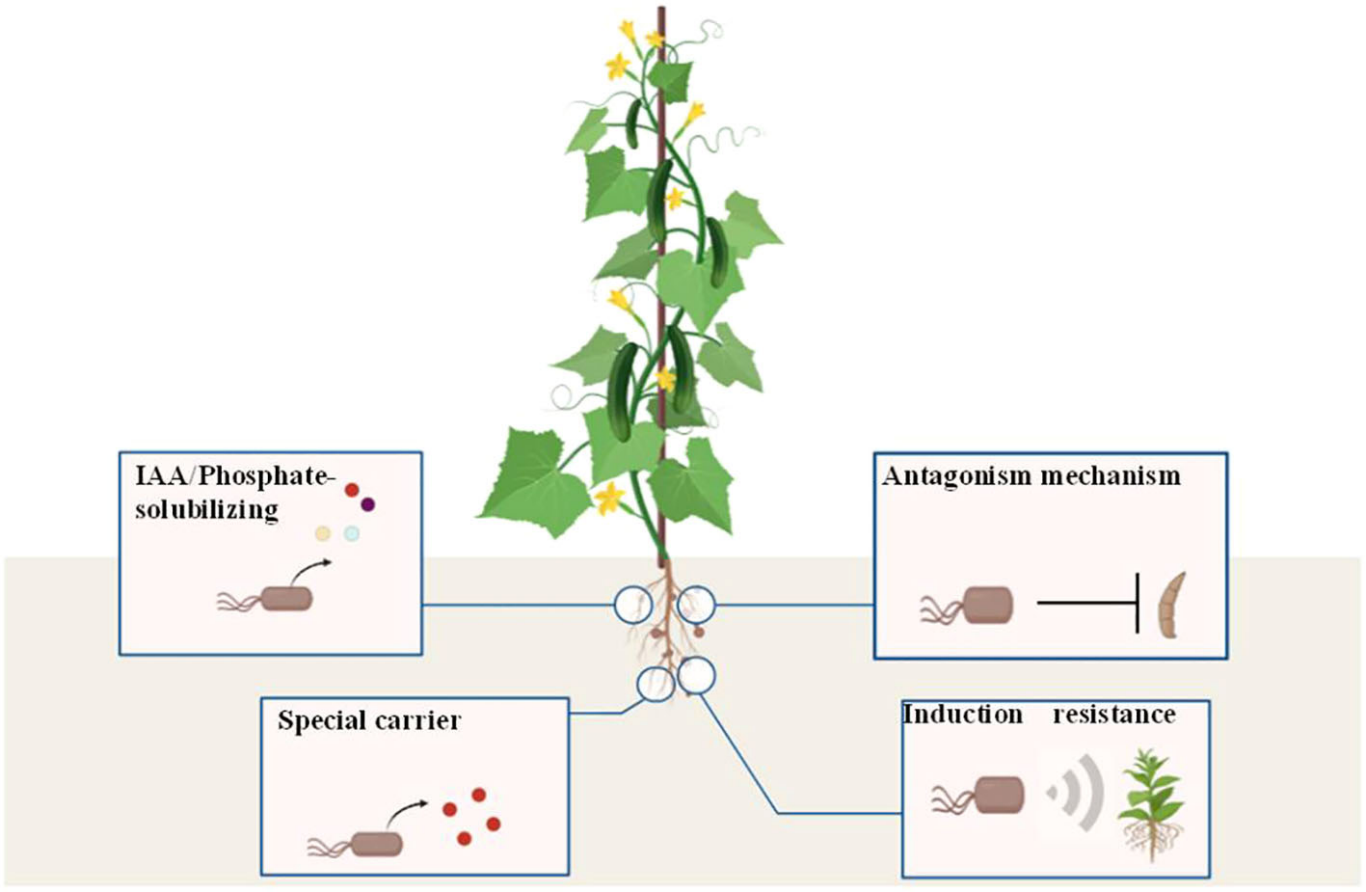
*Bacillus velezensis* strain LJ-19 antagonizing *Fusarium oxysporum* f. sp. *cucumerinum* growth, inducing disease resistance, and stimulating growth. Strain LJ-19 played a role in stimulating cucumber growth by dissolving inorganic phosphorus, producing indole-3-acetic acid and iron carriers. Meanwhile, strain LJ-19 inhibited the hyphal growth of *Fusarium oxysporum* f. sp. *cucumerinum* to suppress its infection and activated the resistance response of cucumber.

Here, we confirmed that *Bacillus velezensis* LJ-19, isolated from the cucumber rhizosphere, exerts dual synergistic effects: potent biocontrol against *Fusarium oxysporum* f. sp. *cucumerinum* via a contact-dependent mechanism and significant promotion of cucumber growth. This discovery mediated by contact-dependent antagonism redefines the functional potential of *Bacillus velezensis*, proving that its biocontrol efficacy is not limited to metabolite secretion but can also rely on direct interaction with pathogens. The results showed that LJ-19’s efficacy depends on direct contact with pathogens (inducing hyphal deformation), while activating plant immunity (up-regulating the activities of defense enzymes and the expression of defense-related genes) and possessing growth-promoting traits (phosphorus solubilization, siderophore production, and IAA synthesis) (**Figure 7**), demonstrating its multifunctional synergy rather than a single functional trait. This understanding provides valuable insights for the rational development of sustainable biocontrol agents in agriculture, offering a green alternative to chemical pesticides for the control of cucumber *Fusarium* wilt while enhancing crop yield.

## Data Availability

The original contributions presented in the study are included in the article/**Supplementary Material**. Further inquiries can be directed to the corresponding authors.

## References

[B1] Abbass K. Qasim M. Z. Song H. Murshed M. Mahmood H. Younis I. (2022). A review of the global climate change impacts, adaptation, and sustainable mitigation measures. Environ. Sci. pollut. Res. Int. 29, 42539–42559. doi: 10.1007/s11356-022-19718-6, PMID: 35378646 PMC8978769

[B2] Ali A. Elrys A. S. Liu L. Iqbal M. Zhao J. Huang X. . (2022). Cover plants-mediated suppression of fusarium wilt and root-knot incidence of cucumber is associated with the changes of rhizosphere fungal microbiome structure-under plastic shed system of north China. Front. Microbiol. 13, 697815. doi: 10.3389/fmicb.2022.697815, PMID: 35444626 PMC9015784

[B3] Bhattacharyya C. Banerjee S. Acharya U. Mitra A. Mallick I. Haldar A. . (2020). Evaluation of plant growth promotion properties and induction of antioxidative defense mechanism by tea rhizobacteria of Darjeeling, India.Sci. Rep. 10 (1), 1–19. doi: 10.1038/s41598-020-72439-z, PMID: 32968101 PMC7511344

[B4] Cao Y. Chen P. Cao X. Hu A. Ye Y. Li P. (2022). *Bacillus velezensis* HNU24 with significant antagonistic activity against Ralstonia solanacearum and promoting plant growth activity. J. Hainan Normal Univ. (Natural Science) 35, 50–56. doi: 10.12051/j.issn.1674-4942.2022.01.008

[B5] Chen D. Hu H. He W. Zhang S. Tang M. Xiang S. . (2022). Endocytic protein Pal1 regulates appressorium formation and is required for full virulence of *Magnaporthe oryzae*. Mol. Plant Pathol. 23, 133–147. doi: 10.1111/mpp.13149, PMID: 34636149 PMC8659611

[B6] Cuéllar-Torres E. A. Aguilera-Aguirre S. Del Carmen Bañuelos-González M. Xoca-Orozco LÁ Ortiz-Basurto R. I. Montalvo-González E. . (2022). Postharvest application effect of agave fructans on anthracnose disease, defense-related enzyme activities, and quality attributes in avocado fruit. Food Sci. Biotechnol. 31, 1411–1421. doi: 10.1007/s10068-022-01135-7, PMID: 36060563 PMC9433478

[B7] Deveau A. Bonito G. Uehling J. Paoletti M. Becker M. Bindschedler S. . (2018). Bacterial-fungal interactions: ecology, mechanisms and challenges. FEMS Microbiol. Rev. 42, 335–352. doi: 10.1093/femsre/fuy008, PMID: 29471481

[B8] Dong Q. Liu Q. Goodwin P. Deng X. Xu W. Xia M. . (2023). Isolation and Genome-Based Characterization of Biocontrol Potential of *Bacillus siamensis* YB-1631 against Wheat Crown Rot Caused by *Fusarium pseudograminearum*. J. Fungi (Basel) 9 (5), 547. doi: 10.3390/jof9050547, PMID: 37233258 PMC10219336

[B9] Dos Santos-Costa D. Alviano-Moreno D. S. Alviano C. S. Riveiro-da Silva A. J. (2022). Extension of solanaceae food crops shelf life by the use of elicitors and sustainable practices during postharvest phase. Food Bioprocess Technology. 15, 249–274. doi: 10.1007/s11947-021-02713-z

[B10] Fazle Rabbee M. Baek K. H. (2020). Antimicrobial activities of lipopeptides and polyketides of *bacillus velezensis* for agricultural applications. Molecules 25, 4973. doi: 10.3390/molecules25214973, PMID: 33121115 PMC7662345

[B11] Gao M. Xiong C. Gao C. Tsui C. K. Wan M. Zhou X. . (2021). Disease-induced changes in plant microbiome assembly and functional adaptation. Microbiome 9, 187. doi: 10.1186/s40168-021-01138-2, PMID: 34526096 PMC8444440

[B12] Gousiadou C. Kouskoumvekaki I. (2018). Computational analysis of LOX1 inhibition identifies descriptors responsible for binding selectivity. ACS Omega. 3, 2261–2272. doi: 10.1021/acsomega.7b01622, PMID: 30023828 PMC6044675

[B13] Ha-Tran D. M. Nguyen T. T. M. Hung S. H. Huang E. Huang C. C. (2021). Roles of plant growth-promoting rhizobacteria(PGPR) in stimulating salinity stress defense in plants: A review. Int. J. Mol. Sci. 22, 3154. doi: 10.3390/ijms22063154, PMID: 33808829 PMC8003591

[B14] Hibbing M. E. Fuqua C. Parsek M. R. Peterson S. B. (2010). Bacterial competition: surviving and thriving in the microbial jungle. Nat. Rev. Microbiol. 8, 15–25. doi: 10.1038/nrmicro2259, PMID: 19946288 PMC2879262

[B15] Keshmirshekan A. De Souza Mesquita L. M. Ventura S. P. M. (2024). Biocontrol manufacturing and agricultural applications of *Bacillus velezensis*. Trends Biotechnol. 42, 986–1001. doi: 10.1016/j.tibtech.2024.02.003, PMID: 38448350

[B16] Klein T. A. Ahmad S. Whitney J. C. (2020). Contact-dependent interbacterial antagonism mediated by protein secretion machines. Trends Microbiol. 28, 387–400. doi: 10.1016/j.tim.2020.01.003, PMID: 32298616

[B17] Kumar N. Ebel R. C. (2016). Oxidative metabolism in ‘Valencia’ Sweet orange (Citrus sinensis osbeck) abscission zone tissue treated with the abscission agent 5-chloro-3-methyl-4-nitro-1H-pyrazole. HortScience 51, 377–382. doi: 10.21273/HORTSCI.51.4.377

[B18] Lau E. T. Tani A. Khew C. Y. Chua Y. Q. San Hwang S. (2020). Plant growth-promoting bacteria as potential bio-inoculants and biocontrol agents to promote black pepper plant cultivation. Microbiological Res. 240, 126549. doi: 10.1016/j.micres.2020.126549, PMID: 32688172

[B19] Li Z. Guo B. Wan K. Cong M. (2015). Effects of bacteria-free filtrate from Bacillusmegaterium strain L2 on the mycelium growth and spore germination of Alternariaalternata. Biotechnol. Biotechnol. Equip. 29, 1062–1068. doi: 10.1080/13102818.2015.1068135

[B20] Li J. Hu M. Xue Y. Chen X. Lu G. Zhang L. . (2020). Screening, identification and efficacy evaluation of antagonistic bacteria for biocontrol of soft rot disease caused by dickeya zeae. Microorganisms 8 (5), 697. doi: 10.3390/microorganisms8050697, PMID: 32397545 PMC7285164

[B21] Li W. Sun L. Wu H. Gu W. Lu Y. Liu C. . (2024). *Bacillus velezensis* YXDHD1–7 prevents early blight disease by promoting growth and enhancing defense enzyme activities in tomato plants. Microorganisms 12, 921. doi: 10.3390/microorganisms12050921, PMID: 38792750 PMC11124510

[B22] Li S. Xiao Q. Yang H. Huang J. Li Y. (2022). Characterization of a new *Bacillus velezensis* as a powerful biocontrol agent against tomato gray mold. Pestic Biochem. Physiol. 187, 105199. doi: 10.1016/j.pestbp.2022.105199, PMID: 36127070

[B23] Lian H. Li R. Ma G. Zhao Z. Zhang T. Li M. (2023). The effect of Trichoderma harzianum agents on physiological-biochemical characteristics of cucumber and the control effect against *Fusarium* wilt. Sci. Rep. 13, 17606. doi: 10.1038/s41598-023-44296-z, PMID: 37848461 PMC10582011

[B24] Lin L. Shen D. Shao X. Yang Y. Li L. Zhong C. . (2025). Soil microbiome bacteria protect plants against filamentous fungal infections via intercellular contacts. Proc. Natl. Acad. Sci. U S A. 122, e2418766122. doi: 10.1073/pnas.2418766122, PMID: 39813250 PMC11762177

[B25] Liu Y. Li Y. Bi Y. Jiang Q. Mao R. Liu Z. . (2021). Induction of defense response against Alternaria rot in Zaosu pear fruit by exogenous L-lysine through regulating ROS metabolism and activating defense-related proteins. Postharvest Biol. Technol. 179, 111567. doi: 10.1016/j.postharvbio.2021.111567

[B26] Luo W. Liu L. Qi G. Yang F. Shi X. Zhao X. (2019). Embedding *Bacillus velezensis* NH-1in microcapsules for biocontrol of cucumber fusarium wilt. Appl. Environ. Microbiol. 85, 13. doi: 10.1128/AEM.03128-18, PMID: 30824441 PMC6495769

[B27] Ma S. Wang Y. Teng W. (2023). *Bacillus velezensis* K-9 as a potential biocontrol agent for managing potato scab. Plant Dis. 107, 3943–3951. doi: 10.1094/PDIS-12-22-2829-RE, PMID: 37337440

[B28] Marilley L. Vogt G. Blanc M. Aragno M. (1998). Bacterial diversity in the bulk soil and rhizosphere fractions of Lolium perenne and Trifolium repens as revealed by PCR restriction analysis of 16S rDNA. Plant Soil 198, 219–224. doi: 10.1023/A:1004309008799

[B29] Martinez-Viveros O. Jorquera M. A. Crowley D. E. Gajardo G. Mora M. L. (2010). Mechanisms and practical considerations involved in plant growth promotion by rhizobacteria. J. Soil Sci. Plant Nutr. 10, 293–319. doi: 10.4067/S0718-95162010000100006

[B30] Meng Q. Jiang H. Hao J. (2016). Effects of Bacillus velezensis strain BAC03 in promoting plant growth. Biol. Control. 6), 18–26. doi: 10.1016/j.biocontrol.2016.03.010

[B31] Meng X. Wang L. Ma B. Wei X. Zhou Y. Sun Z. . (2024). Screening, identification and evaluation of an acidophilic strain of *Bacillus velezensis* B4–7 for the biocontrol of tobacco bacterial wilt. Front. Plant Sci. 15. doi: 10.3389/fpls.2024.1360173, PMID: 38751839 PMC11094357

[B32] Moon J. H. Won S. J. Maung C. E. H. Choi J. H. Choi S. I. Ajuna H. B. . (2021). *Bacillus velezensis* CE100 inhibits root rot diseases (*Phytophthora* spp.) and promotes growth of Japanese cypress (Chamaecyparis obtusa endlicher) seedlings. Microorganisms. 9, 821. doi: 10.3390/microorganisms9040821, PMID: 33924463 PMC8069221

[B33] Nadarajah K. Abdul Rahman N. S. N. (2023). The microbial connection to sustainable agriculture. Plants 12, 2307. doi: 10.3390/plants12122307, PMID: 37375932 PMC10303550

[B34] Naranjo S. E. Ellsworth P. C. Frisvold G. B. (2015). Economic value of biological control in integrated pest management of managed plant systems. Annu. Rev. Entomol. 60, 621–645. doi: 10.1146/annurev-ento-010814-021005, PMID: 25423598

[B35] Nikolakakis K. Amber S. Wilbur J. S. Diner E. J. Aoki S. K. Poole S. J. . (2012). The toxin/immunity network of Burkholderia pseudomallei contact-dependent growth inhibition (CDI) systems. Mol. Microbiol. 84, 516–529. doi: 10.1111/j.1365-2958.2012.08039.x, PMID: 22435733 PMC3331888

[B36] Ortíz-Castro R. Contreras-Cornejo H. A. Macías-Rodríguez L. López-Bucio J. (2009). The role of microbial signals in plant growth and development. Plant Signaling Behav. 4, 701–712. doi: 10.4161/psb.4.8.9047, PMID: 19820333 PMC2801380

[B37] Park H. L. Seo D. H. Lee H. Y. Bakshi A. Park C. Chien Y. C. . (2023). Ethylene-triggered subcellular trafficking of CTR1 enhances the response to ethylene gas. Nat. Commun. 14, 365. doi: 10.1038/s41467-023-35975-6, PMID: 36690618 PMC9870993

[B38] Pu X. Xie B. Li P. Mao Z. Ling J. Shen H. . (2014). Analysis of the defence-related mechanism in cucumber seedlings in relation to root colonization by nonpathogenic Fusarium oxysporum CS-20. FEMS Microbiol. Lett. 355, 142–151. doi: 10.1111/1574-6968.12461, PMID: 24810367

[B39] René C. A. Tugizimana F. Steenkamp P. A. Dubery I. A. Hassen A. I. Labuschagne N. (2020). Rhizobacteria-induced systemic resilience in Sorghum bicolor(L.) Moench against Fusarium pseudograminearum crown rot under drought stress conditions.Biol. Control 151, 104395–104406. doi: 10.1016/j.micres.2019.126388, PMID: 31865223

[B40] Saeed Q. Xiukang W. Haider F. U. Kučerik J. Mumtaz M. Z. Holatko J. . (2021). Rhizosphere bacteria in plant growth promotion, biocontrol, and bioremediation of contaminated sites: A comprehensive review of effects and mechanisms. Int. J. Mol. Sci. 22 (19), 10529. doi: 10.3390/ijms221910529, PMID: 34638870 PMC8509026

[B41] Sana T. G. Berni B. Bleves S. (2016). The T6SSs of *pseudomonas aeruginosa* strain PAO1 and their effectors: beyond bacterial-cell targeting. Front. Cell Infect. Microbiol. 6, 61. doi: 10.3389/fcimb.2016.00061, PMID: 27376031 PMC4899435

[B42] Santoyo G. Urtis-Flores C. A. Loeza-Lara P. D. Orozco-Mosqueda M. D. C. Glick B. R. (2021). Rhizosphere colonization determinants by plant growth-promoting rhizobacteria(PGPR). Biology. 10, 475. doi: 10.3390/biology10060475, PMID: 34072072 PMC8229920

[B43] Shen Y. Shi Z. Zhao J. Li M. Tang J. Wang N. . (2023). Whole genome sequencing provides evidence for *Bacillus velezensis* SH-1471 as a beneficial rhizosphere bacterium in plants. Sci. Rep. 13, 20929. doi: 10.1038/s41598-023-48171-9, PMID: 38017088 PMC10684890

[B44] Shen X. Wang B. Yang N. Zhang L. Shen D. Wu H. . (2021). Lysobacter enzymogenes antagonizes soilborne bacteria using the type IV secretion system. Environ. Microbiol. 23, 4673–4688. doi: 10.1111/1462-2920.15662, PMID: 34227200

[B45] Steffan B. N. Venkatesh N. Keller N. P. (2020). Get physical: bacterial-fungal interactions and their consequences in agriculture and health. J. Fungi (Basel) 6, 243. doi: 10.3390/jof6040243, PMID: 33114069 PMC7712096

[B46] Stoll A. Salvatierra-Martinez R. Gonzalez M. Araya M. (2021). The role of surfactin production by *Bacillus velezensis* on colonization, biofilm formation on tomato root and leaf surfaces and subsequent protection(ISR) against *Botrytis cinerea*. Microorganisms 9, 14. doi: 10.3390/microorganisms9112251, PMID: 34835375 PMC8626045

[B47] Sun X. Xu Z. Xie J. Hesselberg-Thomsen V. Tan T. Zheng D. . (2022). *Bacillus velezensis* stimulates resident rhizosphere Pseudomonas stutzeri for plant health through metabolic interactions. Isme J. 16, 774–787. doi: 10.1038/s41396-021-01125-3, PMID: 34593997 PMC8483172

[B48] Ta Y. Fu S. Liu H. Zhang C. He M. Yu H. . (2024). Evaluation of *Bacillus velezensis* F9 for Cucumber Growth Promotion and Suppression of Fusarium wilt Disease. Microorganisms 12, 1882. doi: 10.3390/microorganisms12091882, PMID: 39338556 PMC11434287

[B49] Toral L. Rodriguez M. Béjar V. Sampedro I. (2018). Antifungal activity of lipopeptides from Bacillus XT1 CECT8661 against Botrytis cinerea. Front. Microbiolog. 9, 1315. doi: 10.3389/fmicb.2018.01315, PMID: 29997581 PMC6028715

[B50] Trivedi P. Batista B. D. Bazany K. E. Singh B. K. (2022). Plant–microbiome interactions under a changing world: responses, consequences and perspectives. New Phytol. 234, 1951–1959. doi: 10.1111/nph.18016, PMID: 35118660

[B51] Wang R. Chen D. Raja A. A. K. Cui J. Hou J. M. Liu T. (2021). A novel Trichoderma asperellum strain DQ-1 promotes tomato growth and induces resistance to gray mold caused by Botrytis cinerea. FEMS Microbiol. Lett. 368, 140. doi: 10.1093/femsle/fnab140, PMID: 34751779

[B52] Wang X. Wang Y. Fu Y. Zhai Y. Bai X. Liu T. . (2024). Multiple omics revealed the growth-promoting mechanism of *Bacillus velezensis* strains on ramie. Front. Plant Sci. 15. doi: 10.3389/fpls.2024.1367862, PMID: 38601307 PMC11004232

[B53] Wang C. Zhao D. Qi G. Mao Z. Hu X. Du B. . (2020a). Effects of *Bacillus velezensis* FKM10 for Promoting the Growth of Malus hupehensis Rehd. and Inhibiting Fusarium verticillioides. Front. Microbiol. 10. doi: 10.3389/fmicb.2019.02889, PMID: 31998247 PMC6965166

[B54] Wang X. Zhou X. Cai Z. Guo L. Chen X. Chen X. . (2020b). A Biocontrol Strain of Pseudomonas aeruginosa CQ-40 Promote Growth and Control Botrytis cinerea in Tomato. Pathogens 10(1), 22. doi: 10.3390/pathogens10010022, PMID: 33396336 PMC7824093

[B55] Wu Q. Wang B. Shen X. Shen D. Wang B. Guo Q. . (2021). Unlocking the bacterial contact-dependent antibacterial activity to engineer a biocontrol alliance of two species from natural incompatibility to artificial compatibility. Stress Biol. 1, 19. doi: 10.1007/s44154-021-00018-x, PMID: 37676524 PMC10441968

[B56] Wu L. Wu H. Chen L. Yu X. Borriss R. Gao X. (2015). Difficidin and bacilysin from Bacillus amyloliquefaciens FZB42 have antibacterial activity against Xanthomonas oryzae rice pathogens. Sci. Rep. 5, 9. doi: 10.1038/srep12975, PMID: 26268540 PMC4534799

[B57] Xie X. Zhang J. Wang H. Lei C. (2021). Research progress of the synthetic and functional mechanisms of natural lipopeptide antibiotics from Bacillus. Chin. J. Antibiot. 46, 362–370. doi: 10.3969/j.issn.1001-8689.2021.05.002

[B58] Xu M. Shi Y. Fan D. Kang Y. Yan X. Wang H. (2023). Co-Culture of White Rot Fungi Pleurotus ostreatus P5 and Bacillus amyloliquefaciens B2: A Strategy to Enhance Lipopeptide Production and Suppress of Fusarium Wilt of Cucumber. J. Fungi(Basel) 9 (11), 1049. doi: 10.3390/jof9111049, PMID: 37998854 PMC10672132

[B59] Xu W. Yang Q. Yang F. Xie X. Goodwin P. H. Deng X. . (2022). Evaluation and genome analysis of *bacillus subtilis* YB-04 as a potential biocontrol agent against *fusarium* wilt and growth promotion agent of cucumber. Front. Microbiol. 13. doi: 10.3389/fmicb.2022.885430, PMID: 35756052 PMC9218633

[B60] Xu T. Zhu T. Li S. Qiao T. (2014). Fungus-inhibitory activity and gene cloning of beta-glucanase from *Bacillus velezensis* YB15. Chin. J. Biol. Control. 30, 276–281. doi: 10.3969/j.issn.2095-039X.2014.02.021

[B61] Xuan Z. Wang Y. Shen Y. Pan X. Wang J. Liu W. . (2024). *Bacillus velezensis* HN-2: a potent antiviral agent against pepper veinal mottle virus. Front. Plant Sci. 15, 1403202. doi: 10.3389/fpls.2024.1403202, PMID: 39049860 PMC11266135

[B62] Yang F. Jiang H. Ma K. Hegazy A. Wang X. Liang S. . (2024). Genomic and phenotypic analyses reveal Paenibacillus polymyxa PJH16 is a potential biocontrol agent against cucumber fusarium wilt. Front. Microbiol. 15, 1359263. doi: 10.3389/fmicb.2024.1359263, PMID: 38591040 PMC11000672

[B63] Yang F. Jiang H. Ma K. Wang X. Liang S. Cai Y. . (2023). Genome sequencing and analysis of *Bacillus velezensis* VJH504 reveal biocontrol mechanism against cucumber Fusarium wilt. Front. Microbiol. 14. doi: 10.3389/fmicb.2023.1279695, PMID: 37901818 PMC10602789

[B64] Ye X. Li Z. Luo X. Wang W. Li Y. Li R. . (2020). A predatory myxobacterium controls cucumber Fusarium wilt by regulating the soil microbial community. Microbiome. 8, 49. doi: 10.1186/s40168-020-00824-x, PMID: 32252828 PMC7137222

[B65] Zavaliev R. Dong X. (2024). NPR1, a key immune regulator for plant survival under biotic and abiotic stresses. Mol. Cell. 84, 131–141. doi: 10.1016/j.molcel.2023.11.018, PMID: 38103555 PMC10929286

[B66] Zhai Q.-H. Pan Z.-Q. Zhang C. Yu H.-L. Zhang M. Gu X.-H. . (2023). Colonization by Klebsiella variicola FH-1 stimulates soybean growth and alleviates the stress of Sclerotinia sclerotiorum. J. Integr. Agric. 22, 2729–2745. doi: 10.1016/j.jia.2023.01.007

[B67] Zhang D. Gao Y. Wang Y. Liu C. Shi C. (2020). Advances in taxonomy, antagonistic function and application of *Bacillus velezensis*. Microbiol. China 47, 3634–3649. doi: 10.13344/j.microbiol.china.190947

[B68] Zhang L. Tan C. Li W. (2024). Phosphorus-, potassium-, and silicon-solubilizing bacteria from forest soils can mobilize soil minerals to promote the growth of rice(*Oryza sativa L.*). Chem. Biol. Technol. Agric. 11, 103. doi: 10.1186/s40538-024-00622-9

[B69] Zhang D. Yu S. Zhao D. Zhang J. Pan Y. Yang Y. . (2021a). Inhibitory effects of non-volatiles lipopeptides and volatiles ketones metabolites secreted by *Bacillus velezensis* C16 against Alternaria solani. Biol. Control 152, 104421. doi: 10.1016/j.biocontrol.2020.104421

[B70] Zhang M. Zhang C. Zhang S. Yu H. Pan H. Zhang H. (2021b). Klebsiella jilinsis 2N3 promotes maize growth and induces resistance to northern corn leaf blight. Biol. Control 156, 104554. doi: 10.1016/j.biocontrol.2021.104554

[B71] Zhou Z. Tang X. Peng L. Ding H. (2023). Complete genome sequence of *Bacillus velezensis* GUAL210, a potential biocontrol agent isolated from pepper rhizosphere. Plant Dis. 107, 915–918. doi: 10.1094/PDIS-07-22-1585-A, PMID: 36265149

[B72] Zhou Y. Wang H. Sun J. Wicaksono W. A. Liu C. He Y. . (2025). Phenazines contribute to microbiome dynamics by targeting topoisomerase IV. Nat. Microbiol. 10, 2396–2411. doi: 10.1038/s41564-025-02118-0, PMID: 40935925

[B73] Zhou Y. Wang H. Xu S. Liu K. Qi H. Wang M. . (2022). Bacterial-fungal interactions under agricultural settings: from physical to chemical interactions. Stress Biol. 2, 22. doi: 10.1007/s44154-022-00046-1, PMID: 37676347 PMC10442017

[B74] Zhou X. Yang Y. Yin Q. Zhang X. Li M. (2021). Application potential of Comamonas testosteroni ZG2 for vegetable cultivation in nickel and cadmium polluted soil. Environ. Technol. Innovation 23, 101626. doi: 10.1016/j.eti.2021.101626

[B75] Zou Q. Niu X. Liu P. Yang H. Chu M. Wang N. . (2023). Growth characteristics of *Bacillus velezensis* antagonistic to *Botrytis cinerea* and its effects on related defense enzyme activities. Sci. Technol. Food Industry. 44, 1–16. doi: 10.13386/j.issn1002-0306.2022060270

