# Supplementary material for: Dual benefits of Bacillus velezensis LJ-19: contact-dependent biocontrol of Fusarium wilt and growth promotion in cucumber
Source: Front Plant Sci. 2025 Dec 11;16:1711383. doi: 10.3389/fpls.2025.1711383 (PMC12738940; doi:10.3389/fpls.2025.1711383)
Supplement: Supplementary file 1 [file Table1.docx]

**Table S1 Oligonucleotide sequences of primers used in qRT-PCR assays**

| ***Gene*** | **Sequence prime** |
| --- | --- |
| ***Actin*** | F: 5^/^-TCCACGAGACTACCTACAACTC-3^/^  R: 5^/^-GCTCATACGGTCAGCGAT-3^/^ |
| ***NPR1*** | F: 5^/^-TTACTGATAAGGGCAAGAAGGCC-3^/^  R: 5^/^-AAAGTTCACAAAGAGCAGGATGG-3^/^ |
| ***PR3*** | F: 5^/^-TGGTCACTGCAACCCTGACA-3^/^  R: 5^/^-AGTGGCCTGGAATCCGACT-3^/^ |
| ***LOX1*** | F: 5^/^-AAGGTTTGCCTGTCCCAAGA-3^/^  R: 5^/^-TGAGTACTGGATTAACTCCAGCCAA-3^/^ |
| ***CTR1*** | F: 5^/^-AAACACGTCGGATAAATATGGCTT-3^/^  R: 5^/^-CATCCATTCAGGCGTTCCAG-3^/^ |
| ***PAL1*** | F: 5^/^-ATGGAGGCAACTTCCAAGGA-3^/^  R: 5^/^-CCATGGCAATCTCAGCACCT-3^/^ |
