# Supplementary material for: Dual benefits of Bacillus velezensis LJ-19: contact-dependent biocontrol of Fusarium wilt and growth promotion in cucumber
Source: Front Plant Sci. 2025 Dec 11;16:1711383. doi: 10.3389/fpls.2025.1711383 (PMC12738940; doi:10.3389/fpls.2025.1711383)
Supplement: Supplementary file 2 [file Table2.docx]

**Table S2 Inhibition of cucumber Fusarium wilt by antagonistic bacterial isolates in dual cultures**

| Isolates No. | Colony diameter（cm） | Inhibition rate（%） |
| --- | --- | --- |
| A15 | 3.2±0.1e | 50.77±1.54f |
| C9 | 3.4±0.1c | 47.69±1.54g |
| C27 | 3.33±0.15d | 49.23±1.54f |
| AC14 | 3.07±0.06f | 52.82±0.89d |
| A8 | 3.6±0.26b | 44.61±4.07g |
| F3 | 3.6±0.1b | 44.62±1.54g |
| A4 | 4.07±0.6a | 37.43±0.0.89h |
| AC7 | 2.6±0.06h | 59.49±0.89b |
| A3 | 2.9±0.01f | 55.38±1.54c |
| A211 | 3.13±0.06e | 51.8±0.89e |
| C1 | 2,67±0.15f | 58.97±2.35b |
| LJ-19 | 1.07±0.12g | 83.59±1.78a |
