# Supplementary material for: Dual benefits of Bacillus velezensis LJ-19: contact-dependent biocontrol of Fusarium wilt and growth promotion in cucumber
Source: Front Plant Sci. 2025 Dec 11;16:1711383. doi: 10.3389/fpls.2025.1711383 (PMC12738940; doi:10.3389/fpls.2025.1711383)
Supplement: Supplementary file 3 [file Table3.docx]

**Table S3 Morphology and colony characteristics of LJ-19 strain**
