# Supplementary figures and images for: Dual benefits of Bacillus velezensis LJ-19: contact-dependent biocontrol of Fusarium wilt and growth promotion in cucumber
Source: Front Plant Sci. 2025 Dec 11;16:1711383. doi: 10.3389/fpls.2025.1711383 (PMC12738940; doi:10.3389/fpls.2025.1711383)

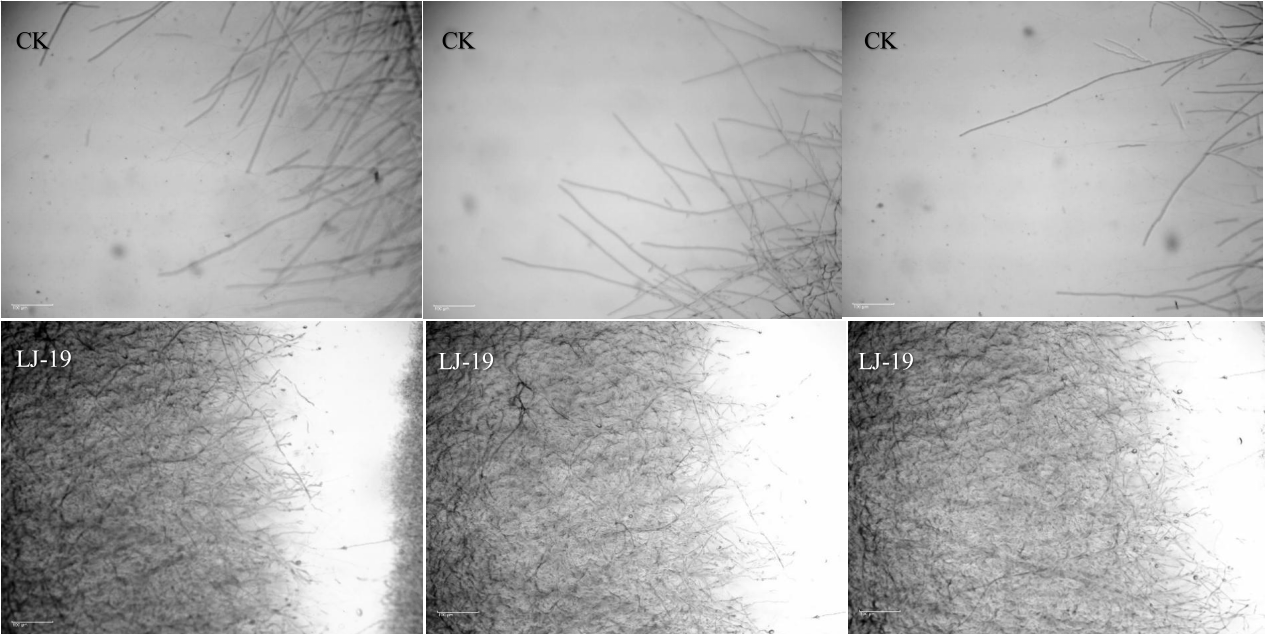


Figure. S1 Different mycelial morphologies of LJ-19

Supplement: Supplementary file 4 [file Table4.docx]
